# Cerebral Excitement Caused by the Bromides

**Published:** 1882-03

**Authors:** 


### CEREBRAL EXCITEMENT CAUSED BY THE BROMIDES.

Dr. Henry M. Bannister, in the Journal of Nervous and Mental Disease, speaks of this peculiar effect of the bromides which he has had the opportunity to witness in one single case, that of a powerfully built man of about thirty years, in robust general health, but liable to frequent attacks of the *grand mal* for twenty-seven years, for which he has repeatedly been treated with the bromides with the effect of stopping his convulsions, but at the same time rendering him liable to attacks of genuine epileptic furore. Being somewhat curious in regard to these facts, Dr. B. resolved to observe the effects of the medicine upon the patient himself, and ordered for him Seguin's prescription of ten grains of the potassium and five grains of the ammonium bromide in an alkaline solution, three times a day. The effect on his general condition was excellent; there were none of the unpleasant phenomena of bromism, not even an acne pimple, so far as observed. The attacks, which had been as frequent as two or three a week, ceased almost entirely, his mind seemed to brighten, he became somewhat more active physically, his functions were all regular, his pulse was all the while normal, circulation and sleep good. But with the general physical and mental improvement in most respects, there gradually appeared an offensive self-importance and quarrelsomeness; and after some three weeks of the treatment he was a very disagreeable and dangerous lunatic; and after he had made an unprovoked assault upon an attendant and had nearly torn the clothes off him it was not considered advisable to continue it any longer. The patient a few days after the discontinuance of the medicine, was the same rational and manageable subject as before, with also the former frequency of his epileptic attacks.

Similar cases having been observed by Drs. Jewell and Kiernan, Dr. B. regards the fact that in these cases the suppression of the epileptic attacks by the bromides was accompanied by cerebral excitement and outbursts of maniacal furore, as strongly suggestive that the attacks themselves are somewhat of the nature of a safety-valve in some cases, and that the epilepsy is itself an alternative to acute and dangerous mania. The cerebral excitement is perhaps not to be ascribed directly to the medicine, but is secondary to its usual therapeutic effect, the suppression of the fits, and this may be the best explanation of the phenomena.—*Med. and Surg. Reporter.*

